# Knowledge, attitudes and practices with regard to schistosomiasis prevention and control: Two cross-sectional household surveys before and after a Community Dialogue intervention in Nampula province, Mozambique

**DOI:** 10.1371/journal.pntd.0007138

**Published:** 2019-02-07

**Authors:** Christian Rassi, Sandrine Martin, Kirstie Graham, Monica Anna de Cola, Celine Christiansen-Jucht, Lauren E. Smith, Ercílio Jive, Anna E. Phillips, James N. Newell, Marilia Massangaie

**Affiliations:** 1 Malaria Consortium, London, United Kingdom; 2 Malaria Consortium, Maputo, Mozambique; 3 Direcção Provincial de Saúde, Nampula, Mozambique; 4 Schistosomiasis Control Initiative, Imperial College, London, United Kingdom; 5 The Nuffield Centre for International Health & Development, University of Leeds, Leeds, United Kingdom; 6 Departamento das Doenças Tropicais Negligenciadas, Ministério de Saúde, Maputo, Mozambique; Universidade Federal de Minas Gerais, BRAZIL

## Abstract

**Background:**

The Community Dialogue Approach is a promising social and behaviour change intervention, which has shown potential for improving health seeking behaviour. To test if this approach can strengthen prevention and control of schistosomiasis at community level, Malaria Consortium implemented a Community Dialogue intervention in four districts of Nampula province, Mozambique, between August 2014 and September 2015.

**Methodology/Principal findings:**

Cross-sectional household surveys were conducted before (N = 791) and after (N = 792) implementation of the intervention to assess its impact on knowledge, attitudes and practices at population level. At both baseline and endline, awareness of schistosomiasis was high at over 90%. After the intervention, respondents were almost twice as likely to correctly name a risk behaviour associated with schistosomiasis (baseline: 18.02%; endline: 30.11%; adjusted odds ratio: 1.91; 95% confidence interval: 1.14–2.58). Increases were also seen in the proportion of people who knew that schistosomiasis can be spread by infected persons and who could name at least one correct transmission route (baseline: 25.74%; endline: 32.20%; adjusted odds ratio: 1.36; 95% confidence interval: 1.01–1.84), those who knew that there is a drug that treats the disease (baseline: 29.20%, endline: 47.55%; adjusted odds ratio: 2.19; 95% confidence interval: 1.67–2.87) and those who stated that they actively protect themselves from the disease and cited an effective behaviour (baseline: 40.09%, endline: 59.30%; adjusted odds ratio: 2.14; 95% confidence interval: 1.40–3.28). The intervention did not appear to lead to a reduction in misconceptions. In particular, the belief that the disease is sexually transmitted continued to be widespread.

**Conclusions/Significance:**

Given its overall positive impact on knowledge and behaviour at population level, Community Dialogue can play an important role in schistosomiasis prevention and control. The intervention could be further strengthened by better enabling communities to take suitable action and linking more closely with community governance structures and health system programmes.

## Author summary

Schistosomiasis is a parasitic neglected tropical disease that affects around 190 million people worldwide, causing chronic ill health and disability. Central to its prevention and control are the acceptance of health interventions such as the distribution of drugs on a mass scale and the adoption of good hygiene and sanitation practices in communities where the disease thrives. One promising method for promoting such behaviours is the Community Dialogue Approach, which involves training volunteers to host regular community meetings, where local health concerns are discussed and culturally appropriate solutions are agreed upon. In 2014/15, Malaria Consortium implemented a Community Dialogue intervention in four districts of Nampula province, Mozambique, to improve knowledge, attitudes and practices with regard to schistosomiasis prevention and control. To assess the effectiveness of the approach, two household surveys were conducted. Results show that before the intervention, knowledge of how schistosomiasis is acquired, transmitted, prevented and treated was low. After the intervention, knowledge and self-reported adoption of positive behaviours had improved substantially, demonstrating that Community Dialogue can play a central role in strengthening disease prevention and control. The approach could be strengthened by further empowering communities to take action and reducing deeply-held misconceptions.

## Introduction

Schistosomiasis is one of the most common parasitic diseases, with approximately 190 million people infected worldwide [1], causing over 100,000 deaths [2] and the loss of over 1.8 million disability-adjusted life years every year [3]. The disease is caused by worms of the *Schistosoma* (*S*.) genus, which use certain types of freshwater snails as intermediary hosts. The infected snails release cercariae, which infect humans by penetrating the skin when they come into contact with freshwater that contains the parasite. Inside the human body, the cercariae go through several stages and eventually grow into adult worms, which live in infected people’s blood vessels. Here they undergo sexual reproduction, producing and releasing eggs into the lumen of the gut, bladder or urinary tract. The parasite’s life cycle is completed when infected people excrete its eggs while urinating or defecating near freshwater, where the eggs hatch and the resultant miracidia infect the snails [4,5]. One of the countries most affected by schistosomiasis is Mozambique, where the main parasite species present is *S*. *haematobium*. Countrywide, the prevalence of *S*. *haematobium* infection among school-age children is 47% [6] and 18 million of the total population of 23.5 million require preventive chemotherapy [7]. *S*. *haematobium* causes urogenital schistosomiasis, of which the defining symptom is blood in the urine. Pathology can also include scarring, calcification, bladder cancer, and occasional ectopic egg granulomas in brain or spinal cord [4,5].

In areas with moderate to high transmission of schistosomiasis, a key disease control strategy is preventive chemotherapy using praziquantel, typically delivered to at-risk populations in the form of periodic, large-scale mass drug administration (MDA) [8]. Other prevention and control strategies recommended by the World Health Organization include increasing access to and use of safe water, improving sanitation and hygiene, and implementing snail control [9]. Individual and community perceptions of schistosomiasis are likely to have a significant impact on schistosomiasis transmission and uptake of disease prevention and control interventions. For example, preventive chemotherapy programmes are more likely to be successful if they are adapted to local circumstances to build trust, address fear of side effects and correct misconceptions about the treatment among target populations [10]. Social and behaviour change and community engagement are therefore crucial elements of successful MDA campaigns [11] and will also have an important role to play in improving adoption of positive health seeking behaviours more generally [12].

The Community Dialogue Approach [13] is a promising social and behaviour change and community engagement intervention, which has shown potential for improving uptake of health services and promoting recommended behaviours in the context of Integrated Community Case Management of childhood illness [14]. In this approach, volunteers from within the community receive a brief training on a health issue and group facilitation skills. Equipped with a set of visual tools, the volunteers then host regular community meetings to discuss the health issue. Each meeting comprises three stages:

1. Explore: community members learn about a health issue and how it affects the community, discussing and resolving doubts and misconceptions;
2. Identify: community members discuss locally applicable solutions to address the health issue;
3. Decide: community members collectively decide what they will do to address the health issue.

The Community Dialogue Approach targets both individual and social behaviour determinants [15]: It seeks to increase individuals’ knowledge and awareness of a health issue by sharing key messages through the volunteers and visual tools (during the “explore” and “identify” stages), but it also aims to influence social norms through public dialogue and collective decision making (during the “identify” and “decide” stages). Although only a small proportion of the population will actively participate in community meetings, knowledge is expected to spread through word of mouth, while changing social norms are expected to affect the wider community to which the norms apply.

To assess if the Community Dialogue Approach can strengthen prevention and control of schistosomiasis at community level, Malaria Consortium conducted an implementation research study in partnership with the Republic of Mozambique’s Ministério de Saúde (Ministry of Health) and the Direcção Provincial de Saúde (Provincial Health Authority) in Nampula province. Between August 2014 and September 2015, 157 volunteers facilitated regular community dialogue meetings in four districts of Nampula province, discussing the causes and symptoms of schistosomiasis and how the disease can be prevented and controlled, including MDA and adoption of improved water, hygiene and sanitation practices. The main tool developed for this intervention was a flipchart containing images illustrating key intervention messages. Approximately 1,500 community meetings were conducted, each typically comprising between 25 and 45 participants.

To evaluate the intervention under programmatic conditions, the implementation research study drew on a range of sources, including qualitative and process evaluation data, mainly focussing on the feasibility and acceptability of the Community Dialogue Approach. Another component of the study explored the intervention’s impact on knowledge, attitudes and practices (KAP) at population level, testing the assumption that the intervention will have impact beyond those who actively participate in community meetings. To this end, two cross-sectional household surveys were conducted in the four study districts, immediately before (baseline) and after (endline) implementation of the Community Dialogue intervention. Baseline survey results have been published [16] and showed that while awareness of the disease was high, correct knowledge of how it is acquired, transmitted and prevented was low. Few respondents reported that their children had received MDA and the misconception that schistosomiasis is sexually transmitted was widespread. Only a minority of respondents reported practicing protective behaviours. This paper presents unpublished endline survey data, demonstrating changes in population-level KAP over the course of the Community Dialogue intervention. A paper describing the intervention in more detail, as well as presenting qualitative and process evaluation data exploring its feasibility and acceptability will be published elsewhere.

## Methods

### Study area

**Fig 1:**
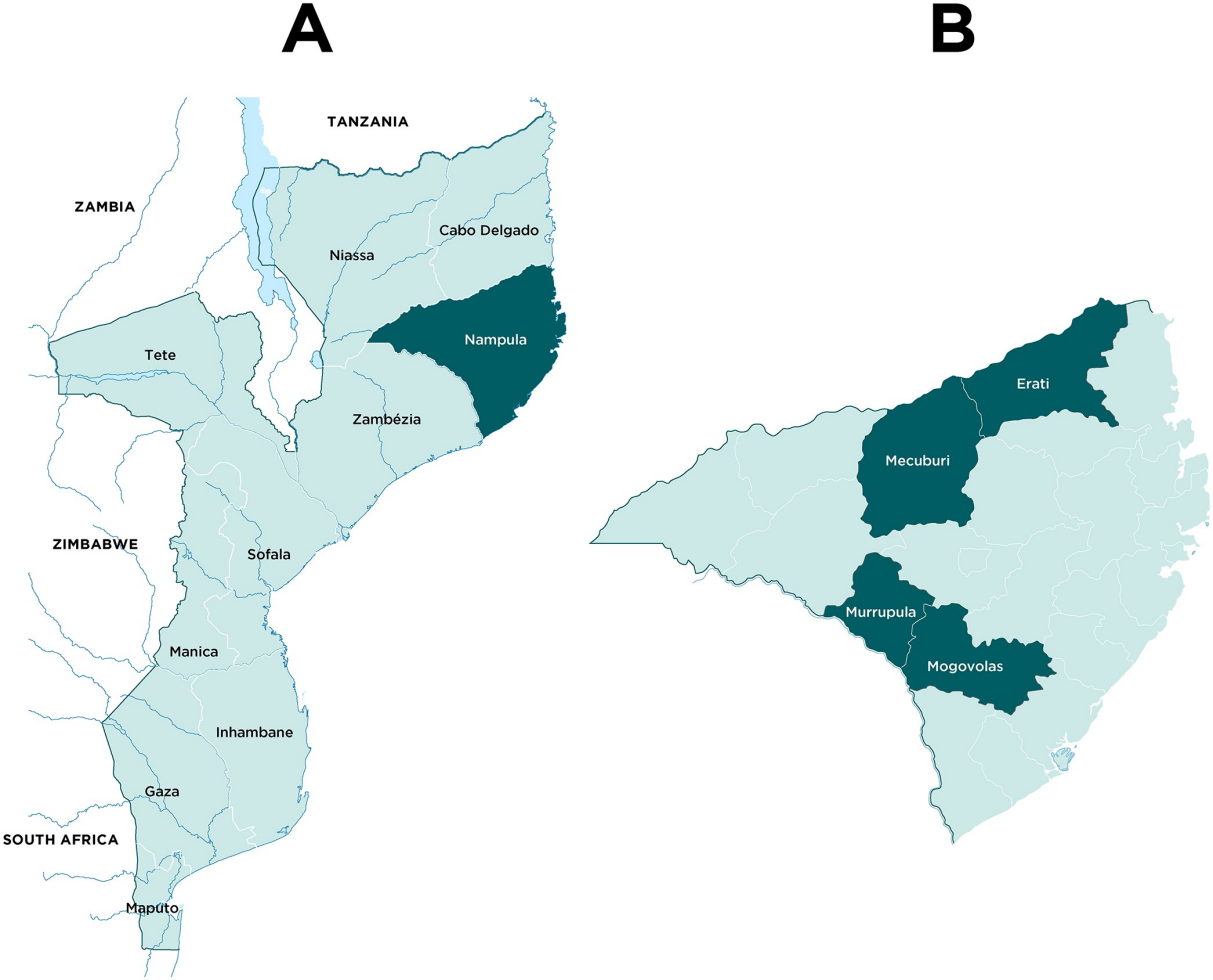
Maps of Mozambique and Nampula province. (A) Location of Nampula province within Mozambique highlighted dark green. (B) Location of intervention districts within Nampula province highlighted dark green.

The study was conducted in Nampula province (Fig 1A), which records the highest provincial schistosomiasis prevalence figures in Mozambique, with around 78% of school-age children infected with *S*. *haematobium* [6]. The four study districts of Eráti, Mecubúri, Mogovolas and Murrupula (Fig 1B) were selected purposively in consultation with the Direcção Provincial de Saúde. Prevalence of schistosomiasis in the districts was known to be high and they were considered to be comparable with regard to the population’s exposure to common risk factors. Specifically, large proportions of the population depend on subsistence agriculture in or near freshwater sources, and there is poor access to clean water for bathing and washing. According to the 2007 Mozambique census, the total population in the four study districts is approximately 839,000 [17].

An integrated neglected tropical disease (NTD) control programme is implemented in all study districts and regular MDA has been conducted since 2009. According to an independent MDA coverage survey carried out in 2015, coverage was comparatively high across northern Mozambique, but lower in Nampula than in any of the other provinces [18]. Only two of the four districts (Mecubúri and Murrupula) received MDA for schistosomiasis while the community dialogue intervention was implemented. These campaigns targeted the entire population over five years of age, whereas MDA conducted before the start of the intervention had targeted school-age children between five and 14 years. Reported programme coverage was 64% and 81% respectively. According to the Direcção Provincial de Saúde, no other programmes targeting schistosomiasis prevention and control were implemented in the study area during the study period.

### Sampling

Baseline and endline KAP surveys were designed as cross-sectional household surveys, with the four study districts considered as one sampling domain. All households were eligible for selection. It was calculated that, when comparing proportions between baseline and endline, a sample size of 389 households was needed to give 80% power to detect a change of at least 10%, conservatively assuming a percentage of 50% at baseline. In order to adjust for confounders, non-response and design effect, the sample size was more than doubled to an intended sample size of 800 households for each survey.

Using proportional-to-size methods, 40 out of the 68 enumeration areas in the four study districts used in the 2007 census were randomly selected. Enumeration areas are based on *localidades*, the lowest level of the central state administration. Some larger *localidades* are further subdivided into *bairros*, which loosely correspond to villages and communities. In a second step, 20 households were selected in each of the 40 enumeration areas, using a random sampling approach adapted from the one recommended for malaria indicator surveys [19]. Household sampling was based on lists of households obtained from community leaders. Taking into account available time and budget, it was not possible to perform household mappings, but field teams were instructed to discuss the reliability of the household lists with community leaders and correct inaccuracies before commencing the sampling process. In each selected household, the household member best placed to answer questions about the household’s health as nominated by the acting household head was interviewed. Only household members over 18 years were eligible. Participation in a Community Dialogue meeting was not a selection criterion. If selected households could not be located, no eligible respondent was available despite at least three repeated visits to the household or respondents declined to be interviewed, the household was dropped from the sample without replacement. To avoid bias and for operational reasons, different samples were selected for the two surveys. It is therefore unlikely that the households sampled or individuals interviewed at baseline and endline were identical.

### Data collection

Baseline data were collected in July 2014, just before the start of the Community Dialogue intervention. The endline survey was conducted in December 2015, shortly after completion of the intervention. On both occasions, data were collected by five field teams, each comprising four researchers and one supervisor. Researchers were typically educated to college level, while supervisors were required to have completed a university degree. All field team members were native speakers of Macua, the language commonly spoken in the study districts. Before the surveys, they were trained on the survey procedures, data collection tools and interview techniques. Consent procedures and issues relating to ethical data collection were also discussed. Researchers were trained for three days and supervisors for four days, including one day in the field to conduct a mock survey.

A structured face-to-face questionnaire (S2 Appendix) was developed in English and subsequently translated into Portuguese. Survey questions were further translated into Macua. Accuracy of the Macua translation was checked with researchers and supervisors during the baseline training. The mock survey conducted as part of the baseline training was used to pre-test the questionnaire. Field teams were instructed to establish the most appropriate local term for schistosomiasis in each community where the survey was conducted in consultation with district health staff and community leaders and to use this term throughout the interview. Otherwise, researchers read out questions exactly as provided. Responses were recorded on paper, assigning them to pre-defined answer categories, which were provided in Portuguese at the request of the field researchers as there is no tradition of reading and writing in Macua. After asking questions about the drug that treats schistosomiasis, field researchers informed all respondents that the name of the drug is praziquantel and showed sample tablets before proceeding to ask whether any of the children living in the household had ever received the drug. Only respondents with children living in their households were asked this question, as prior to the baseline survey, MDA had exclusively targeted school-age children. Table 1 illustrates how the topics covered by the questionnaire relate to the three stages of each Community Dialogue meeting.

**Table 1:** Community Dialogue stages and knowledge, attitudes and practices survey questionnaire topics.

Community Dialogue stage	Questionnaire topic	Explanation
Explore	Awareness of schistosomiasis Knowledge of risk behaviours Knowledge of transmission routes Knowledge of symptoms Knowledge of praziquantel	Communities learn about schistosomiasis from the volunteers with the help of a visual flipchart Participants discuss and resolve misconceptions relating to schistosomiasis Participants exchange experiences of how schistosomiasis affects the community
Identify	Knowledge of prevention and treatment mechanisms	Participants discuss what can be done to strengthen schistosomiasis prevention and control at community level
Decide	Children who have taken praziquantel Self-reported adoption of protective behaviours	Participants publicly commit to a plan of action to adopt positive behaviours

### Data entry and analysis

Data were independently double-entered into EpiData 3.1 (EpiData Association) by data entry officers who had attended a one-day training. Where differences between first and second entry were detected, records were verified against the paper questionnaire. Data were further checked for consistency and prepared for analysis using STATA Version 12 (StataCorp LP). Responses recorded under ‘other’ were reviewed by senior members of the study team and either re-assigned to a pre-defined answer category, assigned to a newly created category or left in the ‘other’ category. The survey procedures in STATA were used to account for the study design. All percentages reported are population average estimates. Only results considered programmatically relevant are reported in this paper, but more detailed survey responses, including 95% confidence intervals (CI), at baseline and endline can be found in S3 Appendix.

To examine the association between baseline and endline KAP results, a multivariate logistic regression analysis was conducted for eleven key indicators. See S4 Appendix for an overview of how the key indicators were operationalised. Odds ratios (ORs) were calculated to provide a quantifiable measure of the increased likelihood of respondents having correct KAP after, compared with before the intervention. ORs were adjusted for sex, education and district, the three socio-demographic characteristics consistently associated with significant KAP differences at baseline [16]. Unadjusted ORs for key indicators are reported in S5 Appendix. Statistical significance of unadjusted and adjusted ORs was determined by a Wald p-value of <0.05.

### Ethics

Ethical approval for the study, including the consent procedures used for the two KAP surveys, was granted by the University of Leeds School of Medicine Research Ethics Committee (SoMREC/13/071) and the Comité Nacional de Bioética para Saúde in Mozambique (42/CNBS/2014). Participation in the surveys was voluntary and informed written consent was taken from all respondents. All data were kept confidential and have been anonymised.

## Results

### Survey respondents

At baseline, five of the 800 randomly selected households could not be located or a suitable respondent was not available despite repeated visits to the household. A further four households were retrospectively excluded from the analysis because respondents’ ages were recorded as under 18. A total of 791 respondents were therefore included in the analysis. At endline, three of the 800 selected households could not be located or a suitable respondent was not available. A further five households were excluded because respondents’ ages were recorded as under 18, resulting in a total of 792 respondents included in the analysis. None of the selected households declined to be interviewed. Table 2 shows survey respondents’ socio-demographic characteristics at baseline and endline.

**Table 2:** Survey respondents’ socio-demographic characteristics at baseline (N = 791) and endline (N = 792).

	Baseline		Endline	
	n	%	N	%
**Sex**				
Female	389	49.18	400	50.51
Male	402	50.82	392	49.49
**Age**				
18–35 years	293	37.04	382	48.23
36–55 years	292	36.92	230	29.04
>55 years	83	10.49	75	9.47
Don’t know	120	15.17	100	12.63
No answer	3	0.38	5	0.63
**Education**				
None	193	24.40	199	25.13
Primary	507	64.10	472	59.60
Secondary	67	8.47	80	10.10
Higher than secondary	14	1.77	39	4.92
Don’t know	4	0.51	2	0.25
No answer	6	0.76	0	0.00
**District**				
Eráti	254	32.11	260	32.83
Mecubúri	139	17.57	141	17.80
Mogovolas	260	32.87	276	34.85
Murrupula	138	17.45	115	14.52

n: number of respondents per socio-demographic category; N: overall sample size.

Adjusted (aOR) and unadjusted ORs for all key indicators were generally found to be similar. There was therefore no confounding by sex, education or district and, with the exception of children who have taken praziquantel, results by socio-demographic respondent characteristics will not be reported in this paper. However, key indicators analysed by sex, education and district can be found in S6 Appendix.

### Awareness and knowledge of schistosomiasis

#### Key indicators

Key indicators with regard to awareness and knowledge of schistosomiasis are shown in Table 3. Awareness of schistosomiasis was high before and after the intervention, with over 90% of respondents at both baseline (91.96%; 95% CI: 89.51–93.89) and endline (91.28%; 95% CI: 88.17–93.63) stating that they had heard of the disease. Note that all subsequently reported results relate to the subsample of those who had heard of schistosomiasis only.

**Table 3:** Sample numbers, population estimates and adjusted odds ratios for key knowledge indicators.

Indicator	Baseline		Endline		Odds ratio^a^	
	n/N	% (95% CI)	n/N	% (95% CI)	Adjusted OR^b^ (95% CI)	Wald p-value
People who have heard of schistosomiasis	721/784	91.96 (89.51–93.89)	722/791	91.28 (88.17–93.63)	0.88 (0.58–1.32)	0.52
People who correctly name at least one risk behaviour^c^	129/716	18.02 (14.63–21.98)	215/714	30.11 (25.05–35.71)	1.91 (1.14–2.58)	<0.01*
People who know that an infected person can contribute to the spread of the disease	338/709	47.67 (42.90–52.49)	294/706	41.64 (37.68–45.71)	0.76 (0.57–1.02)	0.06
People who know that an infected person can contribute to the spread of the disease and can correctly name at least one transmission route^d^	87/338	25.74 (20.30–32.05)	95/295	32.20 (26.76–38.18)	1.36 (1.01–1.84)	0.04*
People who can name at least two effective prevention or treatment mechanisms^e^	91/709	12.83 (9.69–16.80)	108/707	15.28 (11.69–19.72)	1.12 (0.75–1.68)	0.57
People who can correctly name at least two symptoms^f^	485/711	68.21 (64.84–71.41)	474/718	66.02 (61.94–69.87)	0.89 (0.69–1.14)	0.35

n: number of respondents in subsample; N: number of respondents in sample (excluding no answer/missing data); CI: confidence interval; OR: odds ratio.

*denotes a p-value of <0.05.

^a^Reference: Population at baseline for respective indicator.

^b^Odds ratio adjusted for sex, education and district.

^c^Risk behaviours considered correct: fetching contaminated water; poor hygiene/ sanitation habits; bathing/swimming in the river; fishing in infected water; working in rice/agriculture fields.

^d^Transmission routes considered correct: infected person urinating by water; infected person defecating by water.

^e^Prevention or treatment mechanisms considered correct: treat all infected persons; build more latrines/observe better hygiene; treat the water source; treat all people; protect the water source; avoid swimming; use well or pump water.

^f^Symptoms considered correct: blood in urine; painful urination; weight loss; frequent urination; rash/itch; fatigue; fever; swollen stomach; headache; blood in stool; nausea/vomiting; diarrhoea.

Following the intervention, people were almost twice as likely to have correct knowledge of risk behaviours associated with schistosomiasis (aOR: 1.91; 95% CI: 1.14–2.58; p<0.01), with the proportion of respondents who could correctly name at least one risk behaviour increasing from 18.02% (95% CI: 14.63–21.98) at baseline to 30.11% (95% CI: 25.05–35.71) at endline. Conversely, people were less likely (aOR: 0.76; 95% CI: 0.57–1.02; p = 0.06) to know that an infected person can contribute to spreading the disease, though not significantly so. However, among those who knew that schistosomiasis can be spread by infected persons, people were significantly more likely (aOR: 1.36; 95% CI: 1.01–1.84; p = 0.04) to correctly name at least one transmission route at endline (32.20%; 95% CI: 26.76–38.18) than at baseline (25.74%; 95% CI: 20.30–32.05). No significant changes were seen in the likelihood of people citing at least two effective prevention or treatment mechanisms (aOR: 1.12; 95% CI: 0.75–1.68; p = 0.57) or naming at least two symptoms of schistosomiasis (aOR: 0.89; 95% CI: 0.69–1.14; p = 0.35).

#### Knowledge of risk behaviours

Fetching contaminated water (baseline: 9.64%; endline: 11.48%), poor hygiene and sanitation habits (baseline: 7.54%; endline: 9.66%), and bathing or swimming in rivers (baseline: 7.12%; endline: 12.18%) were the most frequently cited correct risk behaviours, with the proportion of respondents mentioning those behaviours all increasing slightly from baseline to endline. The misconception that schistosomiasis can be acquired through sexual contact remained widespread (baseline: 22.07%; endline: 20.59%). There was a noticeable reduction in the proportion of people who stated that they do not know any risk behaviours (baseline: 57.96%; endline: 47.62%).

#### Knowledge of transmission routes

Among respondents who had heard of schistosomiasis and knew that an infected person can spread the disease, knowledge of urinating near freshwater as a transmission route increased (baseline: 23.96%; endline: 29.83%), while knowledge of defecation near freshwater decreased (baseline: 10.36%; endline: 5.42%). Though the proportion of respondents who cited sexual contact as a transmission route decreased slightly (baseline: 81.36%; endline: 75.59%), this misconception remained very common.

#### Knowledge of prevention and treatment mechanisms

Treatment of infected people was the most frequently mentioned correct prevention or treatment mechanism (baseline: 14.23%; endline: 17.26%). The proportion of those who stated that they do not know of any prevention or treatment mechanisms declined noticeably (baseline: 56.76%; endline: 49.50%). However, there was also a decrease in the proportion of respondents citing latrines/good hygiene (baseline: 11.69%; endline: 7.64%). MDA (treatment of all people) was only cited by around 5% of respondents in both surveys (baseline: 5.49%; endline: 5.09%). Misconceptions with regard to avoiding sexual contact remained common, with around a quarter of respondents stating schistosomiasis can be prevented by abstaining from sexual contact with infected persons (baseline: 27.46%; endline: 24.05%).

#### Knowledge of symptoms

The two most commonly cited symptoms of schistosomiasis in both surveys were blood in the urine and painful urination. While knowledge of the former decreased slightly (baseline: 59.44%; endline: 54.95%), knowledge of the latter increased (baseline: 59.30%; endline: 63.93%).

### Mass drug administration and praziquantel

#### Key indicators

Key indicators with regard to MDA and praziquantel are shown in Table 4. Respondents were more than twice as likely to know that there is a drug that treats the disease (aOR: 2.19; 95% CI: 1.67–2.87; p<0.01) after the intervention (47.55%; 95 CI: 43.38–51.75), compared with the baseline (29.20%; 95% CI: 24.72–34.13). The proportion of respondents with children under 18 living in the household who confirmed that at least one of them had taken praziquantel also increased significantly (aOR: 1.62; 95% CI: 1.15–2.28; p<0.01) from 9.33% (95% CI: 6.69–12.86) at baseline to 15.1% (95% CI: 11.30–19.89) at endline. At baseline, 93.33% (95% CI: 90.33–95.45) of respondents with children under 18 living in the household stated that they would want their children to take praziquantel if offered through a treatment campaign. While this proportion remained high at endline (87.12%; 95% CI: 83.67–89.94), it decreased significantly (aOR: 0.44; 95% CI: 0.27–0.70; p<0.01).

**Table 4:** Sample numbers, population estimates and adjusted odds ratios for key indicators with regard to mass drug administration and praziquantel.

Indicator	Baseline		Endline		Odds ratio^a^	
	n/N	% (95% CI)	n/N	% (95% CI)	Adjusted OR^b^ (95% CI)	Wald p-value
People who know there is a drug that treats the disease	205/702	29.20 (24.72–34.13)	339/713	47.55 (43.38–51.75)	2.19 (1.67–2.87)	<0.01*
People with children under 18 living in the household who report that at least one of the children has received praziquantel	54/577	9.33 (6.69–12.86)	77/510	15.10 (11.30–19.89)	1.62 (1.15–2.28)	<0.01*
People with children under 18 living in the household who state that they would want their children to receive praziquantel if offered through a treatment campaign	504/540	93.33 (90.33–95.45)	406/466	87.12 (83.67–89.94)	0.44 (0.27–0.70)	<0.01*

n: number of respondents in subsample; N: number of respondents in sample (excluding no answer/missing data); CI: confidence interval; OR: odds ratio.

*denotes a p-value of <0.05.

^a^Reference: population at baseline for respective indicator.

^b^Odds ratio adjusted for sex, education and district.

#### Children who have taken praziquantel

The proportion of people with children under 18 living in the household who reported that at least one of the children had taken praziquantel increased in three out of the four study districts, most noticeably in Murrupula (baseline: 12.66%; endline: 27.27%), one of the two districts where MDA for schistosomiasis had been conducted during the community dialogue implementation period. However, in Mecubúri, the other district where MDA for schistosomiasis had been implemented, the proportion of people who reported that their children had taken praziquantel decreased slightly (baseline: 13.68%; endline: 12.35%).

### Adoption of protective practices

#### Key indicators

Key indicators with regard to the adoption of protective behaviours associated with schistosomiasis are presented in Table 5. The likelihood of people reporting that they actively do something to protect themselves and their household from the disease increased slightly (aOR: 1.18; 95% CI: 0.87–1.60; p = 0.27). However, after the intervention, people who reported practicing a protective behaviour were more than twice as likely (aOR: 2.14; 95% CI: 1.40–3.28; p<0.01) to cite an effective behaviour. This proportion increased from 40.09% (95% CI: 32.59–48.09) at baseline to 59.30% (95% CI: 50.83–67.26) at endline.

**Table 5:** Sample numbers, population estimates and adjusted odds ratios for key indicators relating to the adoption of protective behaviours.

	Baseline		Endline		Odds ratio^a^	
	n/N	% (95% CI)	n/N	% (95% CI)	Adjusted OR^b^ (95% CI)	Wald p-value
People who report that they do something to protect themselves from the disease	230/677	33.97 (28.57–39.83)	259/680	38.09 (33.52–42.88)	1.18 (0.87–1.60)	0.27
People who report that they do something to protect themselves and cite at least one effective behaviour^c^	89/222	40.09 (32.59–48.09)	153/258	59.30 (50.83–67.26)	2.14 (1.40–3.28)	<0.01*

n: number of respondents in subsample; N: number of respondents in sample (excluding no answer/missing data); CI: confidence interval; OR: odds ratio.

*denotes a p-value of <0.05.

^a^Reference: population at baseline for respective indicator.

^b^Odds ratio adjusted for sex, education and district.

^c^Protective behaviours considered effective: avoid swimming in infested water; use latrine; boil bathing water.

#### Self-reported protective behaviours

The most commonly mentioned effective behaviour was avoiding swimming in infested water (baseline: 26.58%; endline: 46.90%), with a noticeable increase in the percentage of respondents who cited this behaviour at endline. The proportion of those who stated that they protect themselves from schistosomiasis by not having sex or unprotected sex with an infected person reduced noticeably (baseline: 47.30%; endline: 39.53%), though it remained high at just under 40%.

Among those who stated that they do not actively do something to protect themselves from contracting schistosomiasis, more than three quarters stated that they do not know what they can do (baseline: 80.50%; endline: 78.86%).

## Discussion

Previous studies showed that Community Dialogue can bring about changes in social norms and improve health seeking among participants [20]. This study provides good evidence that the approach can also have a positive impact on knowledge at population level. General awareness of schistosomiasis remained universally high at over 90%, possibly as a result of regular MDA campaigns over a number of years. High levels of awareness have been found consistently in other surveys across sub-Saharan Africa [21–25], including one in neighbouring Cabo Delgado province, Mozambique [26]. A recently published systematic review on schistosomiasis KAP in sub-Saharan Africa concluded, however, that high awareness is typically coupled with poor knowledge of causes, prevention and control [27]. This pattern was also found in the four study districts at baseline [16] and it was therefore encouraging to see generally improved knowledge at endline. Specifically, significant improvements were seen for three key components addressed by the Community Dialogue intervention:

- Better knowledge of risk behaviours can enable community members to reduce exposure to risk of infection.
- Increased knowledge of transmission routes can prompt community members to contribute towards reducing the spread of schistosomiasis.
- Improved knowledge of praziquantel as a drug that treats schistosomiasis can reassure communities that effective treatment options are available and improve health seeking behaviour.

It was also noticeable that for many questions exploring respondents’ knowledge, the proportion of those who indicated that they did not know how to answer the question decreased. However, some decreases in specific areas of knowledge were also observed. For example, fewer respondents knew that infected people can spread schistosomiasis. From the responses recorded by field researchers in the ‘other’ category, it was evident that many respondents were not clear about the difference between infection and transmission, which has also been cited by several other studies examining community-level knowledge of schistosomiasis [23,28]. This is a complex distinction requiring fairly sophisticated understanding of the parasite’s transmission cycle, which, moreover, may not influence people’s motivations and resulting behaviour. A more concerning finding was that knowledge of specific prevention and treatment mechanisms decreased, most alarmingly a reduction in knowledge of defecation by freshwater as a transmission route and awareness of the use of latrines and good hygiene as prevention mechanisms. This suggests that training materials and tools developed for the Community Dialogue intervention will need to be reviewed to ensure less well-understood aspects of disease prevention and control are adequately communicated. It also suggests that the “identify” stage of this Community Dialogue intervention, in which participants are encouraged to discuss locally appropriate actions to prevent and control schistosomiasis, needs to be strengthened, for example by providing more detailed and specific guidance with regard to how communities can actively contribute to improved disease prevention and control.

It was also noticeable that only around 5% of respondents cited MDA as a prevention and control mechanism. However, “treatment for all infected people” was frequently cited, which may reflect a misinterpretation of the rationale behind the approach, especially in a context where infection rates are very high. The surveys also found a decrease in the number of respondents who stated that they would want their children to receive praziquantel through MDA, which may be an unintended side effect of strengthening community members’ self-efficacy. It is worth noting, however, that the vast majority of respondents remained supportive of MDA. Nevertheless, the intervention may need to put more emphasis on the safety and community-level benefits of MDA during the “identify” stage.

The overall increase in correct knowledge of schistosomiasis did not appear to lead to a significant reduction in misconceptions. In particular, the belief that the disease is sexually transmitted continued to be widespread. This misconception has been reported by a number of studies in a range of countries [21,22,24] and is perhaps not surprising given that some of the most common symptoms of urogenital schistosomiasis affect the reproductive organs. The persistence of traditional beliefs despite adoption of biomedically correct disease causality models has been well documented, for example with regard to malaria and HIV/AIDS [29,30]. It may be necessary to more directly address known misconceptions in the intervention’s key messages communicated during the “explore” stage, which involves participants’ exchanging experiences and challenging each other’s ideas and concepts with the help of the Community Dialogue volunteers. Training materials and intervention tools should also be reviewed in light of survey findings to ensure they adequately address and do not entrench misconceptions, especially those that might obstruct the adoption of positive behaviours.

Results with regard to the adoption of protective behaviours were mixed. While those who reported actively doing something were significantly more likely to cite an effective behaviour at endline, a majority of around 60% of respondents did not report practicing any protective behaviours. The proportion of those who stated that they do not practice a protective behaviour because they do not know what they can do also remained high at around 80%. It was also concerning that while there was a significant increase in respondents who reported that their children had received praziquantel, this proportion remained very low at 15%, despite MDA campaigns in two of the four study districts while the Community Dialogue intervention was implemented. Two factors may have played a role in limiting the intervention’s population-level impact on behaviour. First, it is well known that improved knowledge does not automatically translate into increased uptake of recommended practices [31], especially where interventions seek to address complex sets of behaviours that are entrenched and structured by poverty, livelihood patterns and social environments [32]. While the Community Dialogue Approach encourages communities to collectively reach decisions about tackling health issues, it is likely that, in many communities, there was an initial focus on increasing knowledge. Second, behaviour change is a non-linear process that unfolds over time [33]. Approximately 14 months of implementing a Community Dialogue intervention may not be sufficient to affect widespread behaviour change.

The limited impact on adoption of positive health seeking behaviours therefore points to a need to strengthen the “identify” and “decide” stages of the Community Dialogue intervention, in which communities were asked to publicly debate how the community could improve schistosomiasis prevention and control and to collectively commit to a plan of action. The intervention could, for example, give more concrete examples of the kind of action people could take, assist planning for and monitoring of actions taken by the community and better embed this process in the existing health system and community governance mechanisms to strengthen social accountability. The intervention should also aim to create stronger links between communities and health care providers or other relevant programmes, such as those addressing water, sanitation and hygiene (WASH), who could assist communities in making decisions and taking action. This could, for example, be informed by the principles of the Enhanced Development Governance model, which has recently been described for a pilot project in Tanzania that sought to strengthen community participation for WASH programmes [34]. Crucially, the Community Dialogue intervention should also link more closely with the national and provincial MDA programme, possibly by giving the volunteers who facilitate the community dialogues a role in planning and delivering campaigns within their community.

### Limitations

The KAP surveys aimed to detect changes in knowledge, attitudes and practices with regard to schistosomiasis prevention and control at population level over the course of a Community Dialogue intervention. They were not designed to:

- Compare KAP among volunteers or active Community Dialogue participants and the wider population. As participation in community meetings was not a selection criterion and only a small proportion of the total adult population will have been active participants, it is unlikely that any participants interviewed will have biased overall levels of KAP.
- Assess the effectiveness of specific intervention components, such as the performance of the Community Dialogue volunteers or the appropriateness of the tools developed for this intervention.
- Measure the intervention’s impact on health outcomes such as disease prevalence or infection intensity.
- Determine the intervention’s impact on uptake of services. In particular, they were not intended as MDA coverage surveys and can only be seen as a very crude measure of the intervention’s impact on MDA coverage. It is unlikely that the MDA campaigns affected knowledge of schistosomiasis as measured by the surveys: only two of the four study districts saw a campaign targeting schistosomiasis between baseline and endline, whereas improvements in knowledge were seen across all districts. Where there were differences between districts, there was no clear trend favouring districts where recent MDA campaigns targeting schistosomiasis had been conducted. Moreover, social mobilisation is typically limited to broadcasting logistical information about target population, dates and distribution modalities of the campaign a few days prior to MDA campaigns via radio spots and loudspeakers mounted on vehicles.

Another limitation relates to the languages used to develop survey tools and to conduct the survey. While the quality of translations of data collection tools and researcher training materials was checked repeatedly, it is possible that subtleties got lost in the translation chain from English to Portuguese to Macua. Similarly, though care was taken to use appropriate local terminology and formative research carried out before the baseline survey concluded that local terms for schistosomiasis broadly concur with the biomedical definition of the disease, it cannot be ruled out that some respondents may have referred to disparate disease concepts.

Finally, responses regarding adoption of practices need to be interpreted bearing in mind that the survey relied on self-reporting. It was not possible to objectively verify survey participants’ responses. In general, social desirability bias may have led respondents to give answers they considered more socially acceptable. While this would have applied at both baseline and endline, the pressure to give socially desirable responses may have been stronger following an intervention designed to shape social norms.

### Conclusion

The survey results are thought to be representative of the population in the four study districts. As the study area shares many characteristics of predominantly rural, resource-poor areas in sub-Saharan Africa and our findings reflect those of other studies in similar settings, the results reported in this paper are thought to have wider applicability. We therefore believe that, given its overall positive impact on knowledge, attitudes and practices, the Community Dialogue Approach can play an important role in affecting positive social and behaviour change, a key requirement for improved disease prevention and control identified by a recent systematic review [27]. The approach goes beyond more established community engagement approaches for NTD control, such as community-directed delivery of MDA [35] or involving community health workers in the detection of suspected NTD cases [36]. Rather than being community-directed or community-based, it is community-owned and has the potential to serve as a platform for community participation, conceived as a process rather than an outcome [37], beyond a single disease or control intervention. More research is needed, however, to investigate the intervention’s mechanisms of impact, trace the spread of information, as well as uncover the conditions under which Community Dialogue can correct misconceptions and trigger sustainable behaviour change. Further research should also include testing of the required intervention dose and reach to determine the number of facilitators required per population unit, the percentage of the population that needs to actively participate in community meetings, or the required number and frequency of community meetings.

## Supporting information

S1 Appendix STROBE checklist. (PDF)

S2 Appendix Questionnaire (English). (PDF)

S3 Appendix Survey responses at baseline and endline. (XLSX)

S4 Appendix Operationalisation of key indicators. (PDF)

S5 Appendix Unadjusted and adjusted odds ratios for key indicators. (XLSX)

S6 Appendix Key indicators analysed by sex, education and district. (XLSX)
