# Supplementary material for: Knowledge, attitudes and practices with regard to schistosomiasis prevention and control: Two cross-sectional household surveys before and after a Community Dialogue intervention in Nampula province, Mozambique
Source: PLoS Negl Trop Dis. 2019 Feb 7;13(2):e0007138. doi: 10.1371/journal.pntd.0007138 (PMC6382216; doi:10.1371/journal.pntd.0007138)
Supplement: S1 Appendix — (PDF) [file pntd.0007138.s001.pdf]

STROBE Statement—Checklist of items that should be included in reports of ***cross-sectional studies***

	Item No	Recommendation
**Title and abstract**	1	(*a*) Indicate the study's design with a commonly used term in the title or the abstract<br><br>Title indicates that results from two cross-sectional household surveys are reported, before and after an intervention
		(*b*) Provide in the abstract an informative and balanced summary of what was done and what was found<br><br>Abstract reports key information about the methodology used and summary of main results
**Introduction**		
Background/rationale	2	Explain the scientific background and rationale for the investigation being reported<br><br>Introduction, paragraphs 1-4
Objectives	3	State specific objectives, including any prespecified hypotheses<br><br>Introduction, paragraph 5
**Methods**		
Study design	4	Present key elements of study design early in the paper<br><br>Introduction, paragraph 5
Setting	5	Describe the setting, locations, and relevant dates, including periods of recruitment, exposure, follow-up, and data collection<br><br>Methods, Study area, paragraphs 1-2<br>Methods, Data collection, paragraphs 1-2
Participants	6	(*a*) Give the eligibility criteria, and the sources and methods of selection of participants<br><br>Methods, Sampling, paragraphs 1-2
Variables	7	Clearly define all outcomes, exposures, predictors, potential confounders, and effect modifiers. Give diagnostic criteria, if applicable<br><br>Methods, Data entry and analysis, paragraph 1
Data sources/ measurement	8*	For each variable of interest, give sources of data and details of methods of assessment (measurement). Describe comparability of assessment methods if there is more than one group<br><br>Methods, Data entry and analysis, paragraph 1<br>Supplementary file S4 Appendix. Operationalisation of key indicators.
Bias	9	Describe any efforts to address potential sources of bias<br><br>Methods, Sampling, paragraphs 1-2
Study size	10	Explain how the study size was arrived at

		Methods, Sampling, paragraph 1
Quantitative variables	11	Explain how quantitative variables were handled in the analyses. If applicable, describe which groupings were chosen and why<br><br>Methods, Data entry and analysis, paragraphs 1-2
Statistical methods	12	(*a*) Describe all statistical methods, including those used to control for confounding<br><br>Methods, Data entry and analysis, paragraphs 1-2
		(*b*) Describe any methods used to examine subgroups and interactions<br><br>Methods, Data entry and analysis, paragraphs 1-2
		(*c*) Explain how missing data were addressed<br><br>Methods, Data entry and analysis, paragraphs 1-2
		(*d*) If applicable, describe analytical methods taking account of sampling strategy<br><br>Not applicable
		(*e*) Describe any sensitivity analyses<br><br>Not applicable
**Results**		
Participants	13*	(a) Report numbers of individuals at each stage of study—eg numbers potentially eligible, examined for eligibility, confirmed eligible, included in the study, completing follow-up, and analysed<br><br>Results, Survey respondents, paragraph 1
		(b) Give reasons for non-participation at each stage<br><br>Results, Survey respondents, paragraph 1
		(c) Consider use of a flow diagram<br><br>Not applicable
Descriptive data	14*	(a) Give characteristics of study participants (eg demographic, clinical, social) and information on exposures and potential confounders<br><br>Results, Survey respondents, Table 2
		(b) Indicate number of participants with missing data for each variable of interest<br><br>Results, Survey respondents, Table 2
Outcome data	15*	Report numbers of outcome events or summary measures<br><br>Methods, Data entry and analysis, paragraphs 1-2
Main results	16	(*a*) Give unadjusted estimates and, if applicable, confounder-adjusted estimates and their precision (eg, 95% confidence interval). Make clear which confounders were adjusted for and why they were included<br><br>Unadjusted estimates are reported in S5 Appendix. Unadjusted and adjusted odds

		ratios for key indicators.<br>Adjusted estimates are reported throughout the Results section and confounders indicated in Table legends.
		(*b*) Report category boundaries when continuous variables were categorized<br><br>Not applicable
		(*c*) If relevant, consider translating estimates of relative risk into absolute risk for a meaningful time period<br><br>Not applicable
Other analyses	17	Report other analyses done—eg analyses of subgroups and interactions, and sensitivity analyses<br><br>Not applicable
**Discussion**		
Key results	18	Summarise key results with reference to study objectives<br><br>Discussion, paragraphs 1-6
Limitations	19	Discuss limitations of the study, taking into account sources of potential bias or imprecision. Discuss both direction and magnitude of any potential bias<br><br>Discussion, Limitations, paragraphs 1-3
Interpretation	20	Give a cautious overall interpretation of results considering objectives, limitations, multiplicity of analyses, results from similar studies, and other relevant evidence<br><br>Discussion, Conclusion
Generalisability	21	Discuss the generalisability (external validity) of the study results<br><br>Discussion, Conclusion
**Other information**		
Funding	22	Give the source of funding and the role of the funders for the present study and, if applicable, for the original study on which the present article is based<br><br>Information about funding has been provided

*Give information separately for exposed and unexposed groups.

Note: An Explanation and Elaboration article discusses each checklist item and gives methodological background and published examples of transparent reporting. The STROBE checklist is best used in conjunction with this article (freely available on the Web sites of PLoS Medicine at http://www.plosmedicine.org/, Annals of Internal Medicine at http://www.annals.org/, and Epidemiology at http://www.epidem.com/). Information on the STROBE Initiative is available at www.strobe-statement.org.
