# Supplementary material for: Knowledge, attitudes and practices with regard to schistosomiasis prevention and control: Two cross-sectional household surveys before and after a Community Dialogue intervention in Nampula province, Mozambique
Source: PLoS Negl Trop Dis. 2019 Feb 7;13(2):e0007138. doi: 10.1371/journal.pntd.0007138 (PMC6382216; doi:10.1371/journal.pntd.0007138)
Supplement: S2 Appendix — (PDF) [file pntd.0007138.s002.pdf]

KAP Survey – Schistosomiasis Prevention and Control in Nampula Province, Mozambique

Section 1: Identification

**Cluster code:** ☐☐	**Household code:** ☐☐
**Interviewer ID:** ☐☐	**Date: (dd/mm/yy)** ______/______/______
**Start time:**	**Respondent's gender:** ☐☐<br>01 = Male<br>02 = Female

Section 2: Interviewee and Household Details

	QUESTIONS AND INSTRUCTIONS	CODING CATEGORIES	SKIP
Q1	**How old are you?**<br><br>SINGLE RESPONSE	0-17 years........ 01<br>18-25 years........ 02<br>26-35 years........ 03<br>36-45 years........ 04<br>46-55 years........ 05<br>56-65 years........ 06<br>Over 65 years........ 07<br><br>Don't know........ 88<br>No answer........ 99	
Q2	**What is your religion?**<br><br>SINGLE RESPONSE	None........ 01<br>Christian (Protestant)........ 02<br>Christian (Roman Catholic)........ 03<br>Christian (Pentecostal)........ 04<br>Christian (Seventh Day Adventist)........ 05<br>Muslim........ 06<br>Hindu........ 07<br>Other........ 08<br><br>Specify:________________________________<br><br>Don't know........ 88<br>No answer........ 99	

	QUESTIONS AND INSTRUCTIONS	CODING CATEGORIES	SKIP
**Q3**	**What is your relationship with the head of this household?**<br><br>SINGLE RESPONSE	Head of household........ 01<br>Spouse........ 02<br>Son/daughter........ 03<br>Stepchild........ 04<br>Adopted/foster child........ 05<br>Son-in-law/daughter-in-law........ 06<br>Grandchild........ 07<br>Parent........ 08<br>Stepparent........ 09<br>Parent-in-law........ 10<br>Brother/sister........ 11<br>Brother-in-law/sister-in-law........ 12<br>Nephew/niece........ 13<br>No relation........ 14<br>Other........ 15<br><br>Specify:____________________<br><br>Don't know........ 88<br>No answer........ 99	
**Q4**	**What is your main occupation?**<br><br>SINGLE RESPONSE	None........ 01<br>Public sector employee (professional)........ 02<br>Public sector employee (manual)........ 03<br>Private sector employee (professional)........ 04<br>Private sector employee (manual)........ 05<br>Self-employed (business)........ 06<br>Self-employed (agriculture/fishery)........ 07<br>Domestic work in household........ 08<br>Religious leader........ 09<br>Teacher........ 10<br>Student........ 11<br>Jobless........ 12<br>Incapacitated........ 13<br>Other........ 14<br><br>Specify:____________________<br><br>Don't know........ 88<br>No answer........ 99	

	QUESTIONS AND INSTRUCTIONS	CODING CATEGORIES	SKIP
Q5	**What is the highest level of education you have completed?**<br><br>SINGLE RESPONSE	None .... 01<br>Started but did not complete primary .... 02<br>Primary .... 03<br>Secondary .... 04<br>*Médio* .... 05<br>Technical course – short duration .... 06<br>Professional course .... 07<br>University .... 08<br>Other .... 12<br><br>Specify:____________________<br><br>Don't know .... 88<br>No answer 99	
Q6	**Does your household have any of the following assets?**<br><br>ASK ABOUT EACH OF THE ASSESTS AND CIRCLE "YES" IF THEY OWN IT OR "NO" IF THEY DO NOT	(see table below)	
Q7	**What sources of water does your household use, for example for drinking or washing clothes?**<br><br>MULTIPLE RESPONSES POSSIBLE<br>CIRCLE ALL MENTIONED<br><br>PROBE TWICE: ANYTHING ELSE?	Water channeled from house to house .... 01<br>Public fountain .... 02<br>Unprotected well .... 03<br>Protected well .... 04<br>Unprotected fountain .... 05<br>Protected fountain .... 06<br>Bore hole .... 07<br>River .... 08<br>Lagoon .... 09<br>Rain water .... 10<br>Bottled water .... 11<br>Tanker-truck .... 12<br>Cistern .... 13<br>Other .... 14<br><br>Specify:____________________<br><br>Don't know .... 88<br>No answer 99	

Q6 coding categories:

	Yes	No	Don't know	No answer
Radio	01	02	88	99
Television	01	02	88	99
DVD player	01	02	88	99
Mobile phone/landline.	01	02	88	99
Motor bike	01	02	88	99

	QUESTIONS AND INSTRUCTIONS	CODING CATEGORIES	SKIP
**Q8**	**What kind of toilet facility do members of your household typically use?**<br><br>SINGLE RESPONSE	Pit latrine........ 01<br>Conventional latrine........ 02<br>Improved latrine........ 03<br>Bush/field........ 04<br>Other........ 05<br><br>Specify:________________<br><br>Don't know........ 88<br>No answer........ 99	

Section 3: Schistosomiasis

	QUESTIONS AND FILTERS	CODING CATEGORIES	SKIP
**Q9**	**Have you heard of schistosomiasis?**<br><br>SINGLE RESPONSE	Yes........01<br>No........02<br><br>Don't know........88<br>No answer........99	 ➔ END<br><br>➔ END<br>➔ END
**Q10**	**In the last six months, where did you hear about it?**<br><br>MULTIPLE RESPONSES POSSIBLE<br>CIRCLE ALL MENTIONED<br><br>PROBE TWICE: ANYTHING ELSE?	Not heard about schistosomiasis in the last six months........01<br>Health professional........02<br>APE........03<br>Treatment campaign........04<br>Community meeting........05<br>School........06<br>Radio/TV........07<br>Newspaper........08<br>Political leader........09<br>Relative/friend/neighbour........10<br>Poster........11<br>Other........12<br><br>Specify:________________<br><br>Don't know........88<br>No answer........99	

	QUESTIONS AND FILTERS	CODING CATEGORIES	SKIP
**Q11**	**How do you get schistosomiasis?**<br><br>MULTIPLE RESPONSES POSSIBLE<br>CIRCLE ALL MENTIONED<br><br>PROBE TWICE: ANYTHING ELSE?	Fetching contaminated water ....01<br>Drinking contaminated water.........................02<br>Fishing in infected water ....03<br>Eating unwashed food ....04<br>Poor hygiene/sanitation habits ....05<br>Bathing/swimming in the river ....06<br>Working in rice/agriculture fields...................07<br>Sexual contact ....08<br>Food/sharing plates..09<br>Urinating in contaminated places ....10<br>Spontaneously/not transmitted ....11<br>Other ....12<br><br>Specify:_________________________________<br><br>Don't know ....88<br>No answer 99	
**Q12**	**Can an infected person contribute towards spreading schistosomiasis?**<br><br>SINGLE RESPONSE	Yes ....01<br>No ....02<br><br>Don't know ....88<br>No answer 99	 → Q14<br><br>→ Q14<br>→ Q14
**Q13**	**How?**<br><br>MULTIPLE RESPONSES POSSIBLE<br>CIRCLE ALL MENTIONED<br><br>PROBE ONCE: ANYTHING ELSE?	Infected person urinating by water................01<br>Infected person defecating by water ....02<br>Infected person has sex/unprotected sex with uninfected person ....03<br>Other ....04<br><br>Specify:_________________________________<br><br>Don't know ....88<br>No answer 99	

	QUESTIONS AND FILTERS	CODING CATEGORIES	SKIP
Q14	**Do you know how you can avoid getting schistosomiasis?**<br><br>MULTIPLE RESPONSES POSSIBLE<br>CIRCLE ALL MENTIONED<br><br>PROBE ONCE: ANYTHING ELSE?	Treatment for all infected persons..................01<br>Treat all people..02<br>Treatment of the water source ....03<br>Protect the water source................................04<br>No swimming..05<br>Use well or pump water ....06<br>More latrines/better hygiene..........................07<br>Don't have sex/unprotected sex with infected person ....08<br>Sexual fidelity/abstinence ....09<br>Others...10<br><br>Specify:_________________________________<br><br>Don't know ....88<br>No answer 99	
Q15	**What are the possible symptoms of schistosomiasis?**<br><br>MULTIPLE RESPONSES POSSIBLE<br>CIRCLE ALL MENTIONED<br><br>PROBE ONCE: ANYTHING ELSE?	Painful urination...1<br>Frequent urination ....2<br>Blood in urine ....3<br>Blood in stool...4<br>Fatigue ....5<br>Fever..6<br>Headache...7<br>Swollen stomach ....8<br>Diarrhea...9<br>Nausea/vomiting ....10<br>Rash/itch..11<br>Weight loss ....12<br>Other ....13<br><br>Specify:_________________________________<br><br>Don't know ....88<br>No answer 99	 <br><br><br><br><br><br><br><br><br><br><br><br><br><br>➔ Q19<br>➔ Q19
Q16	**If you have any of these symptoms, would you seek help?**<br><br>SINGLE RESPONSE	Yes ....01<br>No ....02<br><br>Don't know ....88<br>No answer 99	 ➔ Q18<br><br>➔ Q19<br>➔ Q19

	QUESTIONS AND FILTERS	CODING CATEGORIES	SKIP
Q17	**Who would you ask for help?**<br><br>MULTIPLE RESPONSES POSSIBLE<br>CIRCLE ALL MENTIONED<br><br>PROBE ONCE: ANYONE ELSE?	School teacher ....01<br>Community leader ....02<br>Traditional healer ....03<br>Pharmacy/drug vendor....04<br>APE/ACE/community volunteer ....05<br>Women's group leader....06<br>Religious leader ....07<br>Health worker/health facility ....08<br>Family member....09<br>Other ....10<br><br>Specify:____________<br><br>Don't know ....88<br>No answer 99	→ Q19<br>→ Q19<br>→ Q19<br>→ Q19<br>→ Q19<br>→ Q19<br>→ Q19<br>→ Q19<br>→ Q19<br>→ Q19<br><br><br><br>→ Q19<br>→ Q19
Q18	**Why not?**<br><br>MULTIPLE RESPONSES POSSIBLE<br>CIRCLE ALL MENTIONED<br><br>PROBE ONCE: ANY OTHER REASON?	I don't know who to consult....01<br>I have no money ....02<br>I am not concerned about the symptoms ....03<br>I never seek medical help ....04<br>Other ....05<br><br>Specify:____________<br><br>Don't know ....88<br>No answer 99	
Q19	**Do you know if there is a drug that treats schistosomiasis?**<br><br>SINGLE RESPONSE	Yes, there is ....01<br>No, there isn't....02<br><br>Don't know ....88<br>No answer 99	 → Q22<br><br>→ Q22<br>→ Q22
Q20	**Do you know the name of the drug?**<br><br>SINGLE RESPONSE<br><br>IF RESPONDENT DOES NOT KNOW THE NAME OF THE DRUG, SAY "THE DRUG USED TO TREAT SCHISTOSOMIASIS IS CALLED PRAZIQUANTEL"	Yes, I know the name ....01<br>No, I don't know the name....02<br><br>Don't know ....88<br>No answer 99	 → Q22<br><br>→ Q22<br>→ Q22
Q21	**What is the name of the drug?**<br><br>NOTE RESPONDENT'S ANSWER<br><br>IF RESPONDENT DOES NOT NAME PRAZIQUANTEL, SAY "THE DRUG USED TO TREAT SCHISTOSOMIASIS IS CALLED PRAZIQUANTEL."		

	QUESTIONS AND FILTERS	CODING CATEGORIES	SKIP
**Q22**	SHOW SAMPLE TABLETS<br><br>**This is Praziquantel. It is used for treatment of schistosomiasis. Have you ever taken these tablets?**<br><br>SINGLE RESPONSE	Yes ....01<br>No ....02<br><br>Don't know ....88<br>No answer 99	 ➔ Q27<br><br>➔ Q27<br>➔ Q27
**Q23**	**How did you feel after taking the medication?**<br><br>SINGLE RESPONSE	Felt fine....01<br>Felt slightly unwell....02<br>Felt very unwell ....03<br><br>Don't know ....88<br>No answer 99	
**Q24**	**Have you ever received Praziquantel through a treatment campaign?**<br><br>SINGLE RESPONSE	Yes ....01<br>No ....02<br><br>Don't know ....88<br>No answer 99	➔ Q26<br><br><br>➔ Q27<br>➔ Q27
**Q25**	**Why not?**<br><br>MULTIPLE RESPONSES POSSIBLE<br>CIRCLE ALL MENTIONED<br><br>PROBE ONCE: ANY OTHER REASON?	There was no campaign in my community......01<br>Did not know about the campaign ....02<br>I wasn't offered the drug/the drugs were out-of-stock....03<br>I was not eligible....04<br>I was not ill....05<br>It was not convenient ....06<br>I was scared of side effects....07<br>Drugs don't work....08<br>I prefer traditional medicine ....09<br>Did not trust distributors....10<br>Other ....11<br><br>Specify:______________________<br><br>Don't know ....88<br>No answer 99	➔ Q27<br>➔ Q27<br><br>➔ Q27<br>➔ Q27<br>➔ Q27<br>➔ Q27<br>➔ Q27<br>➔ Q27<br>➔ Q27<br>➔ Q27<br>➔ Q27<br><br><br><br>➔ Q27<br>➔ Q27
**Q26**	**Would you take Praziquantel again if there was another campaign?**<br><br>SINGLE RESPONSE	Yes ....01<br>No ....02<br><br>Don't know ....88<br>No answer 99	➔ Q29<br>➔ Q28<br><br>➔ Q29<br>➔ Q29
**Q27**	**Would you take Praziquantel if offered to you through a treatment campaign?**<br><br>SINGLE RESPONSE	Yes ....01<br>No ....02<br><br>Don't know ....88<br>No answer 99	➔ Q29<br><br><br>➔ Q29<br>➔ Q29

	QUESTIONS AND FILTERS	CODING CATEGORIES	SKIP
Q28	**Why not?**<br><br>MULTIPLE RESPONSES POSSIBLE<br>CIRCLE ALL MENTIONED<br><br>PROBE ONCE: ANY OTHER REASON?	Would only take if I'm ill....01<br>It's not convenient....02<br>Scared of side effects....03<br>Drugs don't work....04<br>I prefer traditional medicine....05<br>Don't trust distributors....06<br>Other....07<br><br>Specify:________________<br><br>Don't know....88<br>No answer....99	
Q29	**How many children under the age of 18 live in your household?**<br><br>SINGLE RESPONSE	None....01<br>One....02<br>Two....03<br>Three....04<br>Four....05<br>Five....06<br>Six....07<br>Seven....08<br>More than seven....09<br><br>Don't know....88<br>No answer....99	→ Q37<br><br><br><br><br><br><br><br><br><br>→ Q37<br>→ Q37
Q30	**Have any of them ever taken Praziquantel?**<br><br>SINGLE RESPONSE	Yes....01<br>No....02<br><br>Don't know....88<br>No answer....99	 → Q35<br><br>→ Q35<br>→ Q35
Q31	**Did any of them ever feel unwell after taking the medication?**<br><br>SINGLE RESPONSE	Yes....01<br>No....02<br><br>Don't know....88<br>No answer....99	
Q32	**Did any of the children in your household receive Praziquantel through a treatment campaign?**<br><br>SINGLE RESPONSE	Yes....01<br>No....02<br><br>Don't know....88<br>No answer....99	→ Q34<br><br><br>→ Q35<br>→ Q35

	QUESTIONS AND FILTERS	CODING CATEGORIES	SKIP
**Q33**	**Why not?**<br><br>MULTIPLE RESPONSES POSSIBLE<br>CIRCLE ALL MENTIONED<br><br>PROBE ONCE: ANY OTHER REASON?	There was no campaign in my community......01<br>Did not know about the campaign ....02<br>They weren't offered the drug/the drugs were out-of-stock ....03<br>They were not eligible ....04<br>They were not ill ....05<br>It was not convenient ....06<br>I was scared of side effects.............................07<br>Drugs don't work ....08<br>I prefer traditional medicine ....09<br>Did not trust distributors................................10<br>Other ....11<br><br>Specify:___________________________________<br><br>Don't know ....88<br>No answer 99	➔ Q35<br>➔ Q35<br><br>➔ Q35<br>➔ Q35<br>➔ Q35<br>➔ Q35<br>➔ Q35<br>➔ Q35<br>➔ Q35<br>➔ Q35<br>➔ Q35<br><br>➔ Q35<br>➔ Q35
**Q34**	**Would you want your children to take Praziquantel again if there was another campaign?**<br><br>SINGLE RESPONSE	Yes ....01<br>No ....02<br><br>Don't know ....88<br>No answer 99	➔ Q37<br>➔ Q36<br><br>➔ Q37<br>➔ Q37
**Q35**	**Would you want your children to receive Praziquantel if offered through a treatment campaign?**<br><br>SINGLE RESPONSE	Yes ....01<br>No ....02<br><br>Don't know ....88<br>No answer 99	➔ Q37<br><br><br>➔ Q37<br>➔ Q37
**Q36**	**Why not?**<br><br>MULTIPLE RESPONSES POSSIBLE<br>CIRCLE ALL MENTIONED<br><br>PROBE ONCE: ANY OTHER REASON?	Children should only receive treatment if they are ill ....01<br>It's not convenient..02<br>Scared of side effects ....03<br>Drugs don't work ....04<br>I prefer traditional medicine ....05<br>Don't trust distributors...................................06<br>Other ....07<br><br>Specify:___________________________________<br><br>Don't know ....88<br>No answer 99	

	QUESTIONS AND FILTERS	CODING CATEGORIES	SKIP
**Q37**	**Do you agree with the following statements?**<br><br>**My household is affected by schistosomiasis.**<br><br>**Schistosomiasis is a matter of concern for me.**<br><br>**Schistosomiasis can have long-term consequences for my health.**<br><br>**Praziquantel is the best cure for schistosomiasis.**<br><br>CIRCLE RESPONDENT'S REPLY FOR EACH OF THE STATEMENTS.	Yes / No / Don't know / No answer<br><br>01 02 88 99<br><br>01 02 88 99<br><br>01 02 88 99<br><br>01 02 88 88	
**Q38**	**Do you do anything to protect you and your household from schistosomiasis?**<br><br>SINGLE RESPONSE	Yes ....01<br>No ....02<br><br>Don't know ....88<br>No answer 99	 → Q40<br><br>→ END<br>→ END
**Q39**	**What do you do?**<br><br>MULTIPLE RESPONSES POSSIBLE<br>CIRCLE ALL MENTIONED<br><br>PROBE ONCE: ANYTHING ELSE?	Avoid swimming or wading in contaminated water ....01<br>Boil drinking water ....02<br>Treat drinking water ....03<br>Boil bathing water ....04<br>Use latrines ....05<br>Don't have sex/unprotected sex with infected person ....06<br>Sexual fidelity/abstinence ....07<br>Other ....08<br><br>Specify:________________<br><br>Don't know ....88<br>No answer 99	→ END<br>→ END<br>→ END<br>→ END<br>→ END<br>→ END<br>→ END<br>→ END<br><br><br><br>→ END<br>→ END
**Q40**	**Why not?**<br><br>MULTIPLE RESPONSES POSSIBLE<br>CIRCLE ALL MENTIONED<br><br>PROBE ONCE: ANY OTHER REASON?	Don't know what I can do....01<br>I'm not concerned about schistosomiasis ....02<br>I don't have money....03<br>It is not practical ....04<br>Other ....05<br><br>Specify:________________<br><br>Don't know ....88<br>No answer 99	

END

DO YOU HAVE ANY QUESTIONS?

THANK YOU FOR YOUR TIME.

Section 4: Final Result

TO BE COMPLETED BY THE FIELD RESEARCHER

**End time:**	**Final result code:** ☐☐<br>01 = Completed<br>02 = Partially completed

Section 5: Post-interview

TO BE COMPLETED BY SUPERVISOR

Supervisor code: ☐☐

Supervisor’s signature:
