# Supplementary material for: Knowledge, attitudes and practices with regard to schistosomiasis prevention and control: Two cross-sectional household surveys before and after a Community Dialogue intervention in Nampula province, Mozambique
Source: PLoS Negl Trop Dis. 2019 Feb 7;13(2):e0007138. doi: 10.1371/journal.pntd.0007138 (PMC6382216; doi:10.1371/journal.pntd.0007138)
Supplement: S4 Appendix — (PDF) [file pntd.0007138.s004.pdf]

S4 Appendix. Operationalisation of key indicators.

The following table lists the key knowledge, attitude and practices indicators reported and how they were operationalised.

Indicator	Questions in questionnaire[a]	Answers
1. People who have heard of schistosomiasis	Q9 Have you heard of schistosomiasis?	Yes
2. People who correctly name at least one risk behaviour	Q11 How do you get schistosomiasis?	At least one of the following: fetching contaminated water; poor hygiene/ sanitation habits; bathing/swimming in the river; fishing in infected water; working in rice/agriculture fields
3. People who know that an infected person can contribute to the spread of the disease	Q12 Can an infected person contribute towards spreading schistosomiasis?	Yes
4. People who know that an infected person can contribute to the spread of the disease and can correctly name at least one transmission route	Q12 Can an infected person contribute towards spreading schistosomiasis?<br>Q13 How?	Yes<br>AND<br>At least one of the following: infected person urinating by water; infected person defecating by water
5. People who can name at least two effective prevention or treatment mechanisms	Q14 Do you know how you can avoid getting schistosomiasis?	At least one of the following: treat all infected persons; build more latrines/observe better hygiene; treat the water source; treat all people; protect the water source; avoid swimming; use well or pump water
6 People who can correctly name at least two symptoms	Q15 What are the possible symptoms of schistosomiasis?	At least two of the following: blood in urine; painful urination; weight loss; frequent urination; rash/itch; fatigue; fever; swollen stomach; headache; blood in stool; nausea/vomiting; diarrhoea

7. People who know there is a drug that treats the disease	Q19 Do you know if there is a drug that treats schistosomiasis?	Yes, there is
8. People with children under 18 living in the household who report that at least one of the children has received praziquantel	Q29 How many children under the age of 18 live in your household?<br><br>Q30 Have any of them ever taken Praziquantel?	At least one<br><br>AND<br><br>Yes
9. People with children under 18 living in the household who state that they would want their children to receive praziquantel if offered through a treatment campaign	Q34 Would you want your children to take Praziquantel again if there was another campaign?<br><br>Q35 Would you want your children to receive Praziquantel if offered through a treatment campaign?	Yes<br><br>OR<br><br>Yes
10. People who report that they do something to protect themselves from the disease	Q38 Do you do anything to protect you and your household from schistosomiasis?	Yes
11. People who report that they do something to protect themselves and cite at least one effective behaviour	Q38 Do you do anything to protect you and your household from schistosomiasis?<br><br>Q39 What do you do?	Yes<br><br>AND<br><br>At least one of the following: avoid swimming in infested water; use latrine; boil bathing water

[a]Refer to S2 Appendix. Questionnaire (English).
